# Decoding the seminal microbial fingerprint and semen quality: insights from the first Greek pilot study

**DOI:** 10.3389/fmicb.2026.1829292

**Published:** 2026-05-29

**Authors:** Despina Vougiouklaki, Eirini Prountzou, Argiris Siatelis, Konstantinos Ladias, Marios Papaparaskevas, Efstathia Tsakali, Monika Polanska, Jan F.M. Van Impe, Dimitra Houhoula

**Affiliations:** 1 Department of Food Science and Technology, Faculty of Food Sciences, University of West Attica, Athens, Greece; 2 3rd Department of Urology, Medical School, National and Kapodistrian University of Athens, “ATTIKON” University Hospital, Chaidari, Greece; 3 Microbiological Center Life Check, Athens, Greece; 4 Department of Chemical Engineering, BioTeC + Chemical and Biochemical Process Technology and Control, KU Leuven, Gent, Belgium

**Keywords:** 16S rRNA, bacterial communities, fingerprint analytical methods, microbiome, semen

## Abstract

**Background:**

Recent evidence suggests that human semen harbors a complex microbiome that may influence semen quality and male fertility. However, comprehensive characterization of bacterial and fungal communities in semen remains limited, particularly using long-read sequencing technologies.

**Methods:**

In this pilot study, 80 semen samples from 80 men were analyzed and classified based on conventional semen-analysis findings into normozoospermic (*n* = 48) and abnormal (*n* = 32) groups. The abnormal group included men with asthenozoospermia and oligoasthenozoospermia. Microbial profiling was conducted using full-length 16S rRNA gene and internal transcribed spacer (ITS) sequencing on the Oxford Nanopore MinION platform.

**Results:**

Phylum-level analysis revealed distinct microbial profiles between groups. Normozoospermic samples were dominated by Bacillota (64.13%), followed by Actinomycetota (14.61%) and Pseudomonadota (12.95%). In contrast, abnormal samples showed reduced Bacillota abundance (2.32%) and enrichment of Campylobacteriota (10.82%). At the genus level, normozoospermic samples were characterized by predominance of Enterococcus, Cutibacterium, Streptococcus, Finegoldia, and Staphylococcus, whereas abnormal samples showed increased abundance of Campylobacter, Stenotrophomonas, and Agrobacterium. Species-level profiling identified *Enterococcus faecalis* as the dominant species in normozoospermic samples (41.3%), while *Campylobacter ureolyticus* predominated in abnormal samples (49.6%). ITS sequencing did not detect fungal DNA in any semen sample, whereas all fungal controls amplified successfully, confirming methodological reliability.

**Conclusion:**

Our findings demonstrate significant differences in seminal microbiome composition between normozoospermic and abnormal semen samples parameters. Alterations at the phylum, genus, and species levels were associated with impaired semen parameters, suggesting a potential link between microbial dysbiosis and male reproductive health. Furthermore, the absence of detectable fungal communities indicates that fungi may be rare or absent in human semen under physiological conditions. This study provides novel insights into the bacterial and fungal landscape of the seminal microbiome using long-read sequencing technology.

## 1 Introduction

Numerous microorganisms such as bacteria, viruses and yeasts have been discovered to cohabitate in the human’s body organ systems (gut, skin, lung and oral cavity) composing the human microbiome (Ursell et al., 2014). Through interactions with the host’s immune system, metabolism and other physiological functions, the microbiome is crucial to preserving human health. The function of microorganisms in other body regions is comparatively less studied than in the stomach (Altmäe et al., 2019). However, new research indicates that the microbiome plays a major role in these habitats underscoring the need for more comprehensive understanding of the human microbiome outside the gut. The male reproductive system has received less attention especially when it comes to studying the identification and characterization of seminal bacterial communities. In the past, the relationship between microorganisms and male infertility has mostly been examined in relation to acute illnesses that impact the male reproductive system (Leelani and Bole, 2023; Lundy et al., 2021). Although many causes of male infertility can be identified, a proportion of cases—estimated to be up to approximately 30%—remain idiopathic (Jungwirth et al., 2012; Agarwal et al., 2015). Yet, infections of male genitourinary tract caused by sexually transmitted diseases (STP), are associated with genital pathogens such as Mycoplasma spp., Ureaplasma spp. and *Chlamydia trachomatis* (Jungwirth et al., 2012). Evidence derived from conventional culture-dependent methodologies has long supported the notion that the seminal environment contains a limited bacterial load, except in cases of established pathological conditions with direct implications for male fertility. In line with this, the majority of culture-based investigations in infertile populations have not identified a consistent or causal relationship between bacterial presence in semen and deviations in conventional semen quality parameters (Neto et al., 2024). Importantly, the detection of microorganisms in semen should not be inherently interpreted as indicative of infection, as the conceptual framework surrounding host–microbe interactions has evolved. It is now widely accepted that commensal microorganisms may inhabit the male reproductive tract, potentially existing in a state of mutualistic or neutral association with the host (Leelani and Bole, 2023). Research has recently expanded to the identification of the semen microbiome and its potential role in male infertility. More than a few bacteria, including *Lactobacillus iners*, *Gardnerella vaginalis*, Escherichia faecalis, *E. coli*, and *Staphylococcus aureus*, have been found to be associated with male infertility as demonstrated using polymerase chain reaction (PCR) (Franasiak and Scott, 2015).

The advent of next-generation sequencing (NGS), which uses the 16 s ribosomal RNA region of the bacterial genome to identify bacteria has enabled more accurate characterization of the human microbiome, and large-scale microbial genome sequences can now be analyzed (Farahani et al., 2020). In the field of andrology, the use of NGS tools to characterize the bacterial communities colonizing the male genital tract has been the driving force behind this change making it possible to investigate the presence of bacterial microbiome in semen samples and the interactions between microbial communities and male reproductive system. These studies have confirmed that semen is not sterile and the microbiome is a rich and diverse community in both infertile and paternally proven men (Altmäe et al., 2019). Given that semen is rich in lipids, saccharides, glycans, inorganic ions, enzymes, nucleic acids, immune components, proteins and peptides (Aalberts and Stout, 2014; Chiasserini et al., 2015; Drabovich et al., 2014; Jodar et al., 2016; Ronquist et al., 2011), it makes sense that microorganisms would thrive there. Indeed, fructose produced by seminal vesicles serves as a nutrient for microorganisms (Javurek et al., 2016).

Studies using NGS to identify and quantify bacterial species present in semen, reported aerobic, facultative anaerobic and strictly anaerobic bacteria and species considered to be opportunistic pathogens (Farahani et al., 2020). According to Hou et al. (2013), Weng et al. (2014) and Baud et al. (2019) there is clustering of semen microbiome and human semen contains a variety of microorganisms that differ significantly between individuals, indicating that each has a distinct and possibly unique bacterial community composition. Numerous Phyla such as Actinobacteria, Bacteroidetes, Firmicutes, Proteobacteria and OD1 (Parcubacteria) are the most abundant tax of the microbiome (Monteiro et al., 2018; Lundy et al., 2021; Zuber et al., 2023). According to Altmäe et al. (2019), semen samples typically contain the following genera: Corynebacterium, Lactobacillus, Streptococcus, Finegoldia, Prevotella, Staphyloccocus, Anaerococcus, Gardnerella, Pseudomonas and Veillonella. Studies haveidentified that Lactobacillus are frequently in great abundance in semen (Hou et al., 2013; Weng et al., 2014; Baud et al., 2019). Conversely, Monteiro et al. (2018) found that the most abundant genera is Enterococcus, whereas Lactobacillus was found in low abundance in semen, still in higher proportion in control group.

Numerous population and lifestyle factors influence the variability in the microbial composition. Indeed, age, ethnicity, diet, body mass index (BMI), diseases, therapies (such as antibiotic, antifungal, and antiviral treatments), administration of pre- and probiotics, stress, physical activity, smoking, and alcohol consumption, among others, are important modulators of semen microbiome (Altmäe et al., 2019). Comparisons between infertile patients and healthy controls, have revealed differences in bacterial composition of these two groups, studying different sperm parameters (Monteiro et al., 2018; Lundy et al., 2021; Osadchiy et al., 2024).

The aim of the present study was to comprehensively characterize the seminal microbiome, including both bacterial and fungal communities, using long-read sequencing technology, and to investigate its association with conventional semen-quality parameters. Furthermore, we aimed to identify distinct microbial patterns (“microbial fingerprint”) associated with normozoospermic and abnormal semen profiles, the latter including asthenozoospermic and oligoasthenozoospermic men.

## 2 Materials and methods

### 2.1 Study design and semen samples collection

Eighty independent semen samples were collected from 80 men following written informed consent. Participants were classified into two groups based on conventional semen-analysis parameters. The normozoospermic group (*n* = 48) comprised healthy donors, including 30 proven fertile men who had fathered at least one child and 18 men with no history of infertility. The abnormal group (*n* = 32) included men with asthenozoospermia or oligoasthenozoospermia who had failed to achieve conception after ≥ 12 months of unprotected intercourse. For the purposes of microbiome analysis, asthenozoospermic and oligoasthenozoospermic samples were combined into a single “abnormal” group to enable comparison with normozoospermic controls. Exclusion criteria included antibiotic use within the preceding 3 months, evidence of male accessory gland infection (negative seminal culture), and sexually transmitted infections (syphilis, HIV, hepatitis B/C).

Participants were instructed to maintain sexual abstinence for 4 days prior to sample collection. Semen samples were obtained by masturbation in a dedicated sample collection room and collected into sterile plastic containers under aseptic conditions. Following liquefaction, samples were incubated at 37 °C and analyzed within 30–60 min after collection. Semen analysis was performed in accordance with the World Health Organization (WHO) Laboratory Manual for the Examination and Processing of Human Semen (6th edition, 2021). Sperm concentration and motility were determined using a Sqaiq Clinical Sperm Quality Analyzer. Sperm motility was validated by microscopic examination of wet preparations, while sperm vitality was evaluated by counting stained (non-viable) and unstained (viable) spermatozoa and expressed as percentages. Seminal pH was measured using pH indicator paper and compared against a calibrated reference strip.

Based on spermiogram findings, samples were classified into mutually exclusive phenotypic groups. Normozoospermia was defined by normal semen parameters. Asthenozoospermia was characterized by reduced sperm motility (total motility < 42% and/or progressive motility < 30%), whereas oligoasthenozoospermia was defined by reduced sperm concentration (< 16 million/mL) combined with decreased sperm motility. Accordingly, samples were categorized as normozoospermic controls (Group C, *n* = 48), asthenozoospermic (Group AT, *n* = 10), and oligoasthenozoospermic (Group OA, *n* = 22).

### 2.2 DNA extraction

Genomic DNA was extracted using the Magcore^®^ automated bacterial DNA extraction kit (Magcore), according to the manufacturer’s instructions. DNA was eluted in nuclease-free buffer, quantified using the Qubit™ dsDNA High Sensitivity Assay Kit (Thermo Fisher Scientific), and stored at −20 °C until further analysis. DNA was eluted in nuclease-free buffer, quantified using the Qubit™ dsDNA High Sensitivity Assay Kit (Thermo Fisher Scientific), and stored at −20 °C until further analysis.

All sample handling and DNA extraction procedures were performed under aseptic conditions using sterile, nuclease-free consumables to minimize the risk of contamination. PCR setup was conducted in a dedicated clean area following standard good laboratory practices. Three fungal isolates were included as positive controls to validate amplification efficiency and downstream sequencing performance.

### 2.3 Amplicon-based 16S rRNA and ITS sequencing

Bacterial and fungal community profiling was performed by targeted amplification of the full- length 16S rRNA gene, spanning all hypervariable regions (V1–V9), and the internal transcribed spacer (ITS) region, respectively.

For each sample, 10 ng of genomic DNA was used as input for PCR amplification. Amplifications were carried out separately for each target using primers provided with the Microbial Amplicon Barcoding Sequencing Kit (SQK-MAB114.24, Oxford Nanopore Technologies).

PCR reactions were performed in a total volume of 50 μL, containing genomic DNA, primer mix, and LongAmp^®^ Hot Start Taq 2 × Master Mix (New England Biolabs). Thermal cycling conditions consisted of an initial denaturation at 95 °C for 1 min, followed by 30 cycles of denaturation at 95 °C for 20 s, annealing at 55 °C for 30 s, and extension at 65 °C for 2 min, with a final extension at 65 °C for 5 min. Amplicons were briefly centrifuged and quantified using a Qubit fluorometer.

Amplicon barcoding was performed using unique barcode adapters, with one barcode assigned per sample. Depending on the number of samples processed, 10 ng of each amplicon was used per barcoding reaction. Barcoded amplicons were pooled and purified using AMPure XP magnetic beads at a 0.8 × bead-to-sample ratio, followed by elution in Elution Buffer. The purified library was quantified prior to sequencing adapter attachment.

Rapid sequencing adapters were ligated to the pooled library according to the manufacturer’s instructions. Sequencing libraries were loaded onto R10.4.1 MinION flow cells following standard priming procedures. Sequencing was initiated using MinKNOW software.

### 2.4 Basecalling, bioinformatics, and statistical analysis

Basecalling was performed using the Guppy basecalling agent (version 6.3.7) embedded within the EPI2ME platform (version 5.2.13), converting raw FAST5 files into FASTQ format. Barcode sequences were trimmed and only reads with a minimum quality score (Q-score) of 9 were retained for downstream analysis. Filtered FASTQ files were subsequently aligned using Minimap2. The reliability of subsampled read numbers was verified prior to full taxonomic analysis. Three randomly selected samples from the Oxford Nanopore Technologies (ONT) sequencing output were subsampled to 30,000 and 100,000 reads per sample to assess sequencing depth adequacy. Negligible differences (<0.1%) were observed across all taxonomic levels, indicating that a sequencing depth of 30,000 reads per sample was sufficient for detecting minor bacterial constituents. For the main taxonomic analysis, 50,000 reads per sample were used to further ensure robust coverage. Alpha diversity rarefaction curves were generated to confirm adequate sequencing depth and to verify the plateauing of diversity indices. Statistical analyses were performed using GraphPad Prism 10 for macOS, while data visualization and graphical representation were carried out using SRplot (Jodar et al., 2016).

## 3 Results

For downstream microbiome analyses, samples were grouped into two categories: normozoospermic (control) and abnormal semen samples, the latter including both asthenozoospermic and oligoasthenozoospermic participants.

### 3.1 Clinical and demographic characteristic of the samples

The clinical, demographic, and semen characteristics of the study population are presented in Table 1. The mean age of the participants was 32.0 ± 1.66 years (range: 27–42 years), with no statistically significant difference between normozoospermic and abnormal groups. All participants were of Greek ethnicity. Regarding body mass index (BMI), the majority of participants had normal BMI values, and no significant differences were observed between groups. In contrast, significant differences were observed in key semen parameters. Normozoospermic samples showed significantly higher sperm concentration, total motility, progressive motility, viability, and normal morphology compared to the abnormal group (*p* < 0.001 for all). No statistically significant differences were observed in semen volume or pH between groups.

**Table 1:** Baseline demographic and semen characteristics of study participants stratified by group, including WHO reference values.

Parameter	WHO criteria	Normozoospermic (*n* = 48)	Abnormal (*n* = 32)	*p*-value
Age (years)	–	31.5 ± 1.5	32.7 ± 1.8	0.06
BMI (kg/m^2^)	–	23.0 ± 1.8	24.1 ± 2.0	0.8
Volume (mL)	≥1.4	2.2 ± 1.0	1.9 ± 0.9	0.12
pH	≥7.2	7.9 ± 0.4	8.1 ± 0.5	0.07
Sperm concentration (million/mL)	≥16	27.5 ± 3.0	12.5 ± 2.8	<0.001
Total motility (%)	≥42	52.0 ± 3.5	30.0 ± 4.0	<0.001
Progressive motility (%)	≥30	38.0 ± 3.0	20.5 ± 3.5	<0.001
Viability (%)	≥54	65.0 ± 2.5	50.0 ± 3.0	<0.001
Normal morphology (%)	≥15	15.5 ± 1.0	9.0 ± 2.5	<0.001

Data are presented as mean ± standard deviation (SD).

### 3.2 Evaluation of microbial diversity

#### 3.2.1 Phylum-level comparison between normozoospermic and abnormal semen groups

At the phylum level, distinct compositional differences were observed between normozoospermic and abnormal semen samples.

In the normozoospermic group (*n* = 48), the seminal microbiome was predominantly composed of Bacillota, accounting for 64.13% of the total relative abundance, followed by Actinomycetota (14.61%), Pseudomonadota (12.95%), and Bacteroidota (5.77%). Lower relative abundance was detected for Mycoplasmatota (2.54%), while Campylobacteriota and Deinococcota were not detected in normozoospermic samples.

In contrast, the abnormal group (*n* = 32) exhibited a markedly different phylum-level profile. Campylobacteriota emerged as the dominant phylum (48.71%), followed by Pseudomonadota (8.38%) and Bacillota (2.32%). Actinomycetota (0.36%) and Deinococcota (0.11%) were detected at low relative abundances, whereas Bacteroidota and Mycoplasmatota were not observed in the abnormal group.

**Figure 1:**
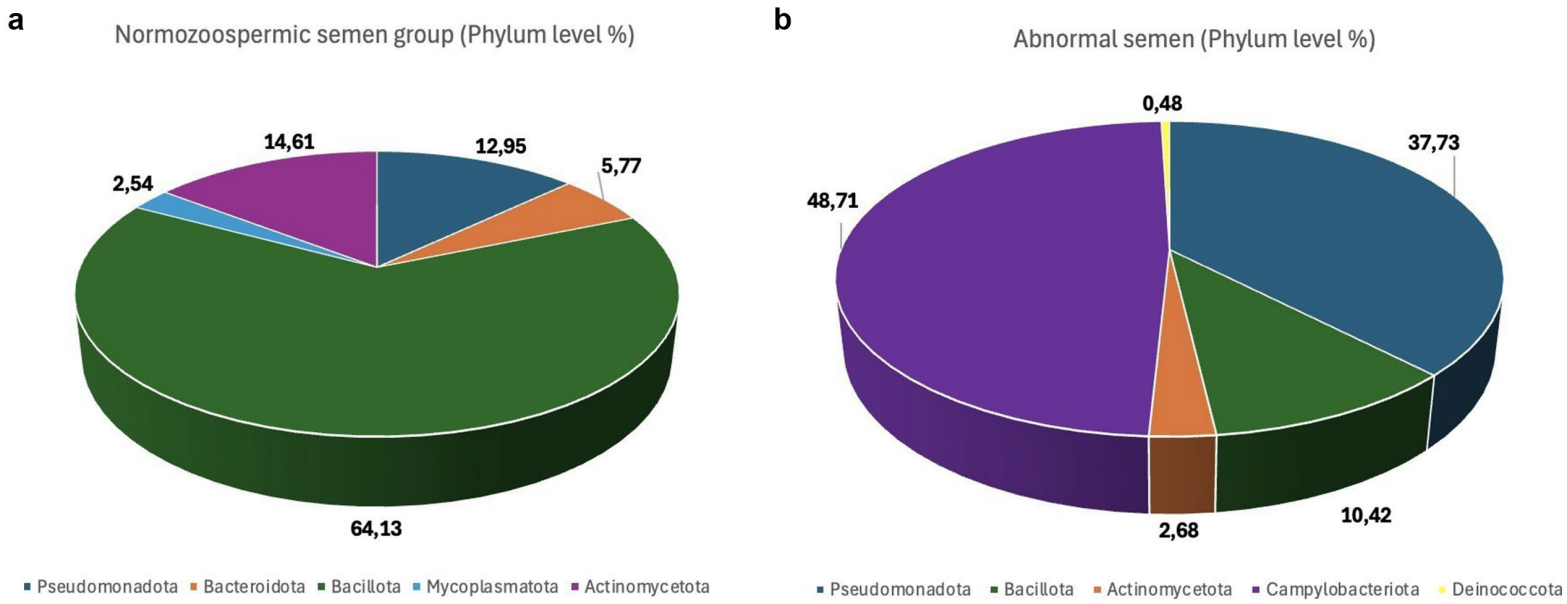
**(a)** Relative abundance (%) of bacterial phyla in normozoospermic semen samples. **(b)** Relative abundance (%) of bacterial phyla in semen samples with abnormal parameters.

Overall, phylum-level analysis demonstrated a clear shift in microbial composition between normal and abnormal semen groups, characterized by reduced representation of Bacillota and relative enrichment of Campylobacteriota in samples with abnormal semen parameters (see Figure 1).

#### 3.2.2 Genus-level comparison between normozoospermic and abnormal semen groups

Genus-level taxonomic profiling revealed further differences in microbial community structure between normozoospermic and abnormal semen samples. In the normozoospermic group, the seminal microbiome was primarily dominated by genera commonly associated with commensal and facultative anaerobic bacteria, including Enterococcus, Cutibacterium, Streptococcus, Finegoldia, and Staphylococcus. These genera collectively constituted the majority of the bacterial community, indicating a relatively homogeneous microbial profile.

In contrast, abnormal semen samples showed increased relative abundance of genera associated with opportunistic and environmental microorganisms, including Campylobacter, Stenotrophomonas, Agrobacterium, Dialister, and Lactococcus. The predominance of Campylobacter in this group was particularly pronounced and contributed substantially to the observed inter-group differences.

**Figure 2:**
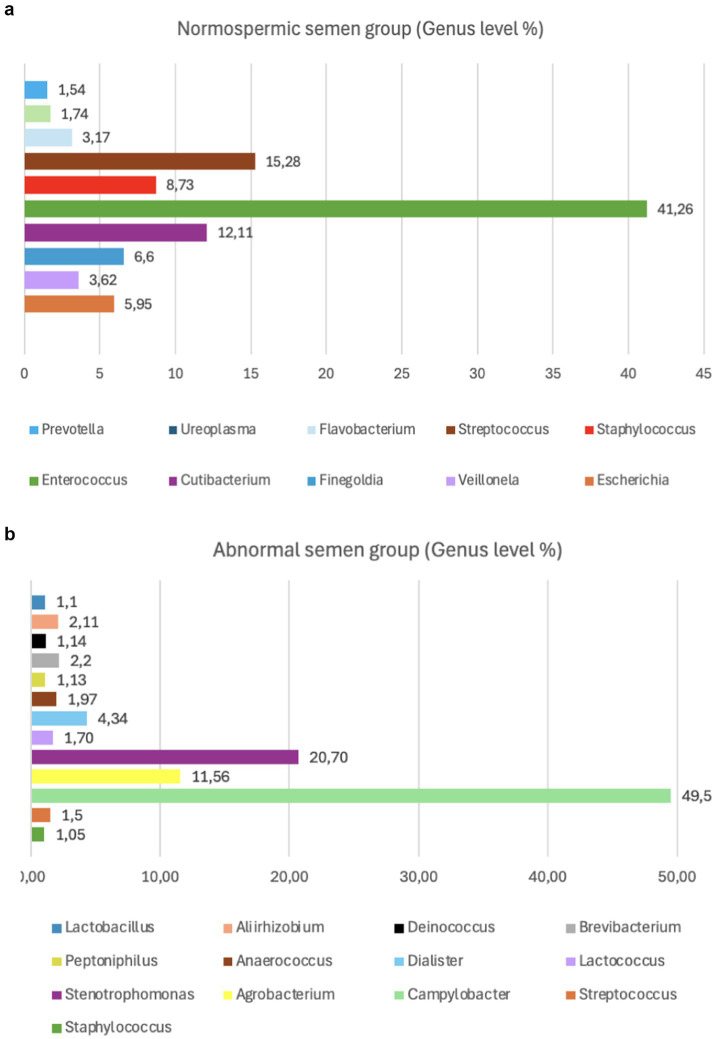
**(a)** Relative abundance (%) of bacterial genus in normozoospermic semen samples. **(b)** Relative abundance (%) of bacterial genus in semen samples with abnormal parameters.

Overall, genus-level analysis indicated a shift from commensal-dominated microbial communities in normozoospermic samples toward more heterogeneous and potentially dysbiotic profiles in samples with abnormal semen parameters (see Figure 2).

#### 3.2.3 Species-level analysis of normozoospermic and abnormal semen samples

Species-level taxonomic profiling was performed on samples meeting quality and read-depth thresholds (*n* = 48 normozoospermic, *n* = 32 abnormal). Distinct compositional patterns were observed between the two groups.

In the normozoospermic group, the seminal microbiome was dominated by *Enterococcus faecalis*, which accounted for approximately 41.3% of the total relative abundance. Other prevalent species included *Cutibacterium acnes* (12.1%), *Streptococcus shenyangsis* (7.1%), *Finegoldia magna* (6.6%), *Staphylococcus hominis* (5.5%), *Escherichia fergusonii* (4.0%), *Streptococcus oralis* (4.6%), *Streptococcus toyakuensis* (3.6%). Collectively, these taxa represented the major constituents of the bacterial community in normozoospermic samples.

**Figure 3:**
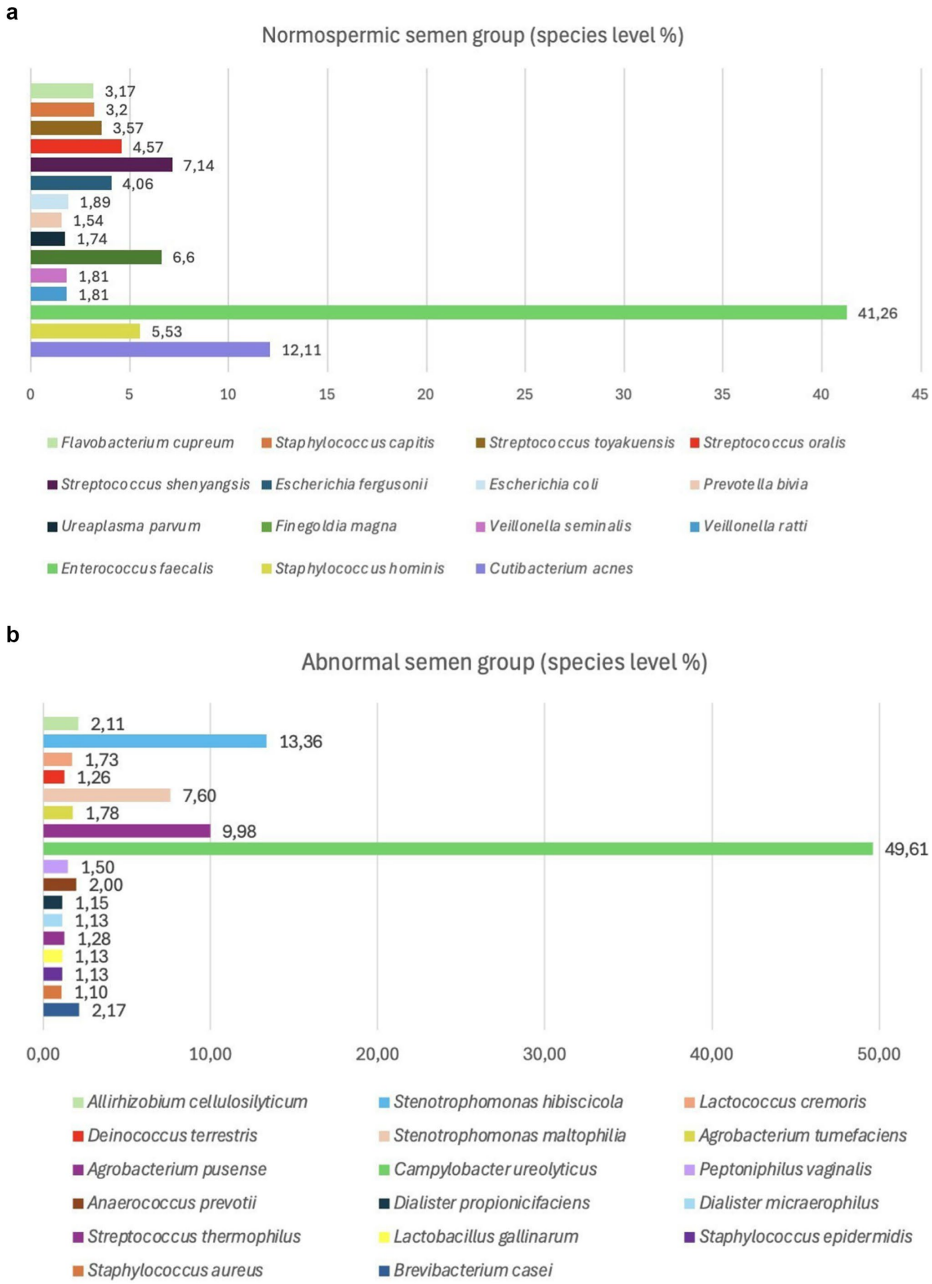
**(a)** Relative abundance (%) of bacterial species in normozoospermic semen samples. **(b)** Relative abundance (%) of bacterial species in semen samples with abnormal parameters.

In contrast, abnormal semen samples showed a markedly different species-level profile, characterized by pronounced dominance of *Campylobacter ureolyticus* (49.6%). Additional species detected at notable relative abundances included Stenotrophomonas hibiscicola (13.4%), Agrobacterium pusense (9.9%), *Stenotrophomonas maltophilia* (7.6%), Lactococcus cremoris (1.7%), *Dialister propionicifaciens* (1.2%), *Anaerococcus prevotii* (2.0%), and Allirhizobium cellulosilyticum (2.1%). Overall, species-level analysis demonstrated substantial differences in microbial composition between normozoospermic and abnormal semen groups, reflecting pronounced inter-group heterogeneity within the seminal microbiome (see Figure 3).

### 3.3 Fungal community analysis

All seminal samples were subjected to fungal community profiling using ITS sequencing. No fungal DNA was detected in any of the samples analyzed. To ensure methodological validity, three distinct fungal control samples were included in the sequencing run. Successful ITS amplification and taxonomic identification were obtained for all control samples, confirming appropriate run performance and excluding technical failure as a cause for the absence of fungal signals in semen samples. These findings suggest that fungal DNA is either absent or present at levels below the detection threshold in the analyzed semen samples.

## 4 Discussion

An increasing body of evidence indicates that human semen harbors a complex and dynamic microbiome, the composition of which is associated with semen quality and male fertility. Studies employing 16S rRNA gene sequencing across diverse populations and sequencing platforms have consistently identified distinct microbial signatures linked to both normal and abnormal spermiogram parameters.

Monteiro et al. (2018) were among the first to demonstrate differences in seminal microbial composition between fertile and infertile men, reporting a predominance of *Enterococcus* and *Staphylococcus*, along with enrichment of *Neisseria*, *Klebsiella*, *Pseudomonas*, *Aerococcus*, and members of the *Enterobacteriaceae* family in men presenting seminal hyperviscosity and oligoasthenoteratozoospermia. Notably, *Lactobacillus* was detected at very low relative abundance across all semen phenotypes. Subsequently, Baud et al. (2019) described three major seminal microbiota profiles—*Prevotella*-dominant, *Lactobacillus*-dominant, and polymicrobial—highlighting that microbial community structure, rather than total bacterial load, distinguishes normal from abnormal semen samples. In that study, *Prevotella*-enriched profiles were associated with impaired semen parameters, whereas *Staphylococcus* and *Lactobacillus* were more frequently observed in normozoospermic samples.

Further evidence from the United States supported these associations. Lundy et al. (2021) reported increased abundance of *Aerococcus* in infertile men and identified an inverse relationship between *Prevotella* abundance and sperm concentration. In contrast, *Pseudomonas* abundance was positively associated with total sperm count, underscoring the functional heterogeneity of bacterial taxa within the seminal environment. Studies focusing on infertility phenotypes and assisted reproduction outcomes provided additional insights (Ribas-Maynou et al., 2021). Okwelogu et al. (2021) observed enrichment of *Lactobacillus* and *Gardnerella* in normozoospermic samples, whereas azoospermic samples showed higher relative abundance of *Mycoplasma* and *Ureaplasma*. Importantly, semen samples associated with successful *in vitro* fertilization were enriched in *Lactobacillus jensenii* and *Faecalibacterium* and depleted of *Proteobacteria*, *Prevotella*, and *Bacteroides*.

Mechanistic associations between seminal microbiota and sperm function were further explored by Garcia-Segura et al. (2022), who reported significant correlations between specific bacterial genera and sperm functional parameters. Increased abundance of *Moraxella*, *Brevundimonas*, and *Flavobacterium* was negatively correlated with sperm DNA fragmentation, while *Brevundimonas* was positively associated with sperm motility and reduced oxidative–reduction potential. Previous studies have also highlighted the clinical relevance of sperm DNA integrity in male fertility and assisted reproductive outcomes (Ribas-Maynou et al., 2021). More recent large-cohort studies reinforced these observations. Vajpeyee et al. (2024) identified significant differences in the distribution of *Lactobacillus* and *Prevotella* between normozoospermic and abnormal semen groups, with reduced *Lactobacillus* prevalence and increased *Prevotella* abundance in men with abnormal spermiograms. Similarly, Osadchiy et al. (2024) reported species-level associations between the seminal microbiome and sperm parameters, including differential abundance of *Lactobacillus iners* and multiple *Pseudomonas* species in relation to sperm motility and concentration. Finally, Campbell et al. (2023) examined men with non-obstructive azoospermia and observed distinct taxonomic shifts compared to fertile controls, including altered proportions of Proteobacteria, Firmicutes, and Actinobacteria, with significant differences in genera such as Escherichia/Shigella, Sneathia, and Raoultella. These findings are consistent with recent systematic reviews, including Neto et al. (2024), which highlight the association between seminal microbiome composition and semen quality, as well as the variability of microbial profiles across populations and methodologies. Recent systematic reviews further support the association between seminal microbiome composition and semen quality.

In particular, Neto et al. (2024) conducted a comprehensive review of available sequencing-based studies and reported that alterations in the seminal microbiome are consistently associated with impaired sperm parameters, including reduced motility and sperm concentration. The authors highlighted that beneficial or commensal taxa, such as Lactobacillus and certain Firmicutes, tend to be enriched in normozoospermic samples, whereas dysbiotic profiles characterized by increased abundance of opportunistic or pathogenic genera, including Proteobacteria and anaerobic bacteria, are more frequently observed in men with abnormal semen parameters.

In the present study, phylum- and species-level profiling further supports the growing evidence that seminal microbiome composition differs between normozoospermic and abnormal semen samples. Normozoospermic samples were characterized by a predominance of *Bacillota*, with *Enterococcus faecalis* representing the most abundant species. This finding is consistent with previous reports identifying *Enterococcus* as a common constituent of the healthy seminal microbiome (Monteiro et al., 2018; Campbell et al., 2023). In contrast, samples with abnormal semen parameters exhibited a marked enrichment of *Campylobacteriota*, driven primarily by the dominance of *Campylobacter ureolyticus*. This observation aligns with studies reporting increased prevalence of potentially pathogenic or opportunistic taxa in semen associated with impaired sperm parameters (Garcia-Segura et al., 2022; Osadchiy et al., 2024).

Notably, the abnormal semen group displayed increased representation of environmental and opportunistic bacterial genera, including *Stenotrophomonas* and *Agrobacterium*, suggesting a potential association between dysbiosis and altered seminal conditions. Although the limited sample size precludes causal inference, the pronounced inter-group compositional differences observed here reinforce the hypothesis that shifts in the seminal microbiome are linked to semen quality.

Importantly, fungal community profiling using ITS sequencing revealed no detectable fungal DNA in any of the semen samples analyzed. Successful amplification and taxonomic identification of fungal control samples confirmed the robustness of the methodology, indicating that the absence of fungal signatures reflects a true biological finding rather than technical failure. To our knowledge, this is among the first studies to systematically assess the seminal mycobiome using ITS-based sequencing, providing novel evidence that fungi may be absent or present at extremely low abundance in human semen under physiological conditions.

## 5 Limitations

This study has several limitations that should be acknowledged. Although key exclusion criteria were applied, additional factors known to influence the seminal microbiome and semen quality such as smoking status, systemic inflammatory conditions, prostatitis, antifungal use, and recent febrile illness—were not systematically recorded; therefore, residual confounding cannot be excluded. Furthermore, the cross-sectional design of the study precludes any conclusions regarding causality between microbiome composition and semen quality. In addition, the relatively limited sample size and the classification of participants based on semen parameters should be considered when interpreting the findings. Future studies with larger sample sizes should investigate microbiome differences across specific semen abnormality subgroups to further refine and validate these findings.

## 6 Conclusion

This study provides a comprehensive characterization of the seminal microbiome in a Greek cohort using long-read sequencing technology. Significant differences in microbial composition were observed between normozoospermic men and men with abnormal semen parameters at the phylum, genus, and species levels. Normozoospermic samples were dominated by commensal-associated taxa, whereas abnormal samples showed enrichment of potentially dysbiotic and opportunistic microorganisms, particularly *Campylobacter ureolyticus*. Notably, the absence of detectable fungal DNA in all analyzed semen samples represents an important finding of this study. Overall, these results demonstrate a clear association between seminal microbiome composition and semen quality.

## Data Availability

The raw data supporting the conclusions of this article will be made available by the authors, without undue reservation.
